# Paper-based triboelectric nanogenerators and their applications: a review

**DOI:** 10.3762/bjnano.12.12

**Published:** 2021-02-01

**Authors:** Jing Han, Nuo Xu, Yuchen Liang, Mei Ding, Junyi Zhai, Qijun Sun, Zhong Lin Wang

**Affiliations:** 1 Beijing Institute of Nanoenergy and Nanosystems, Chinese Academy of Sciences, Beijing, 101400, P. R. China; 2 School of Nanoscience and Technology, University of Chinese Academy of Sciences, Beijing, 100049, P. R. China; 3 Center on Nanoenergy Research, School of Physical Science and Technology, Guangxi University, Nanning, 530004, P. R. China; 4 Qichen (Shanghai) Medical Co., Ltd., Shanghai 201319, P. R. China; 5 College of Materials Science and Engineering, Changsha University of Science & Technology, Changsha, 410114, P. R. China; 6 School of Materials Science and Engineering, Georgia Institute of Technology, Atlanta, Georgia 30332-0245, United States

**Keywords:** energy harvesting, interaction, Internet of Things (IoT), paper-based sensors, self-powered devices, P-TENGs, triboelectric nanogenerator

## Abstract

The development of industry and of the Internet of Things (IoTs) have brought energy issues and huge challenges to the environment. The emergence of triboelectric nanogenerators (TENGs) has attracted wide attention due to their advantages, such as self-powering, lightweight, and facile fabrication. Similarly to paper and other fiber-based materials, which are biocompatible, biodegradable, environmentally friendly, and are everywhere in daily life, paper-based TENGs (P-TENGs) have shown great potential for various energy harvesting and interactive applications. Here, a detailed summary of P-TENGs with two-dimensional patterns and three-dimensional structures is reported. P-TENGs have the potential to be used in many practical applications, including self-powered sensing devices, human–machine interaction, electrochemistry, and highly efficient energy harvesting devices. This leads to a simple yet effective way for the next generation of energy devices and paper electronics.

## Introduction

Environmental pollution is an undeniable fact in our daily lives. The air pollution caused by industrial waste generation (gases/toxins) and by the combustion of fossil fuels is getting more and more serious [1–2]. Meanwhile, with the rapid growth of the Internet of Things (IoTs), the explosive growth of sensors has led to the massive use of batteries, which have also resulted in severe environmental issues in virtue of their short lifetime. In this regard, renewable energy sources, such as wind, wave, and solar power, appear to be the most efficient and effective solutions [3–10]. However, the infrastructure constructions for harvesting energy from renewable sources (e.g., wind power stations and solar photovoltaic energy systems) are huge, expensive, and take a long time to be built. Even worse, harvested wind and solar energy cannot be incorporated into the power grid, which inevitably calls for additional energy storage facilities [11–13]. Therefore, there are still increasing demands for the development of power sources which are highly efficient, clean, and sustainable.

In recent years, the triboelectric nanogenerator (TENG), first invented by the Wang group in 2012 [14], has been quickly developed to be a revolutionary breakthrough in the energy harvesting [15–21] and self-powered systems [22–27]. Based on electrostatic induction and triboelectrification [28], the novel TENG can utilize the Maxwell’s displacement current to readily drive electrons to flow through an external circuit and power portable electronic devices. To harvest the ubiquitous mechanical energy from its surroundings, TENGs need to have a simple device design and to be low cost and lightweight. TENGs have also shown the pivotal ability to convert low-frequency mechanical energy from walking, waving, and eye-blinking into electricity. TENGs can readily serve as a sustainable power supply based on four basic operation modes [29], including vertical contact–separation mode [30–32], lateral-sliding mode [33–35], single-electrode mode [36–37], and freestanding triboelectric-layer mode [38]. As an advanced and durable energy source, TENGs have shown promising and significant features that are applied to power units in the micro- and nanoscale [39–44], high-voltage sources [45], self-powered systems [46–50], and blue energy harvesting devices [51–56].

Paper, by far one of the most inexpensive and flexible materials widely used in daily life, was developed more than 2000 years ago in China. Paper and other fiber-based materials are integral components of many objects that are used on a regular basis by the population, which are also available in different compositions, thickness and surface roughness. Most importantly, paper is biocompatible, biodegradable, and environmentally friendly, and has tremendous advantages over the majority of other materials (e.g., it is lightweight, renewable, and air-permeable). Besides, paper is flexible and can be easily folded or bent into 3D structures without causing structural damage.

In the last decades, paper-based electronic devices, such as microfluidic paper-based analytical devices (µPADs) [57–60] and thin-film transistors (TFTs) [61–65] have been widely investigated. Recently, they have also been applied in various energy-related devices [66–68]. Although paper is intrinsically insulating, conductive materials (e.g., metal nanowires, conducting polymers, carbon nanotube (CNT) inks, multiwall carbon nanotube (MWCNT) inks, and reduced graphene oxide) [69–82], can be easily absorbed or used as a coating layer on the surface of the paper due to its wettability and moisture-retention capacity. This provides an efficient method to prepare paper electrodes for TENGs. Paper has also been proven to be a natural TENG friction layer. Due to that, it shows a tendency of easily losing electrons (i.e., electropositive) when contacting a material that can easily gain electrons (i.e., electronegative). Furthermore, due to the high roughness and porous nanofiber structure it can lead to enhanced TENG output performances owing to improved charge-trapping abilities.

**Figure 1:**
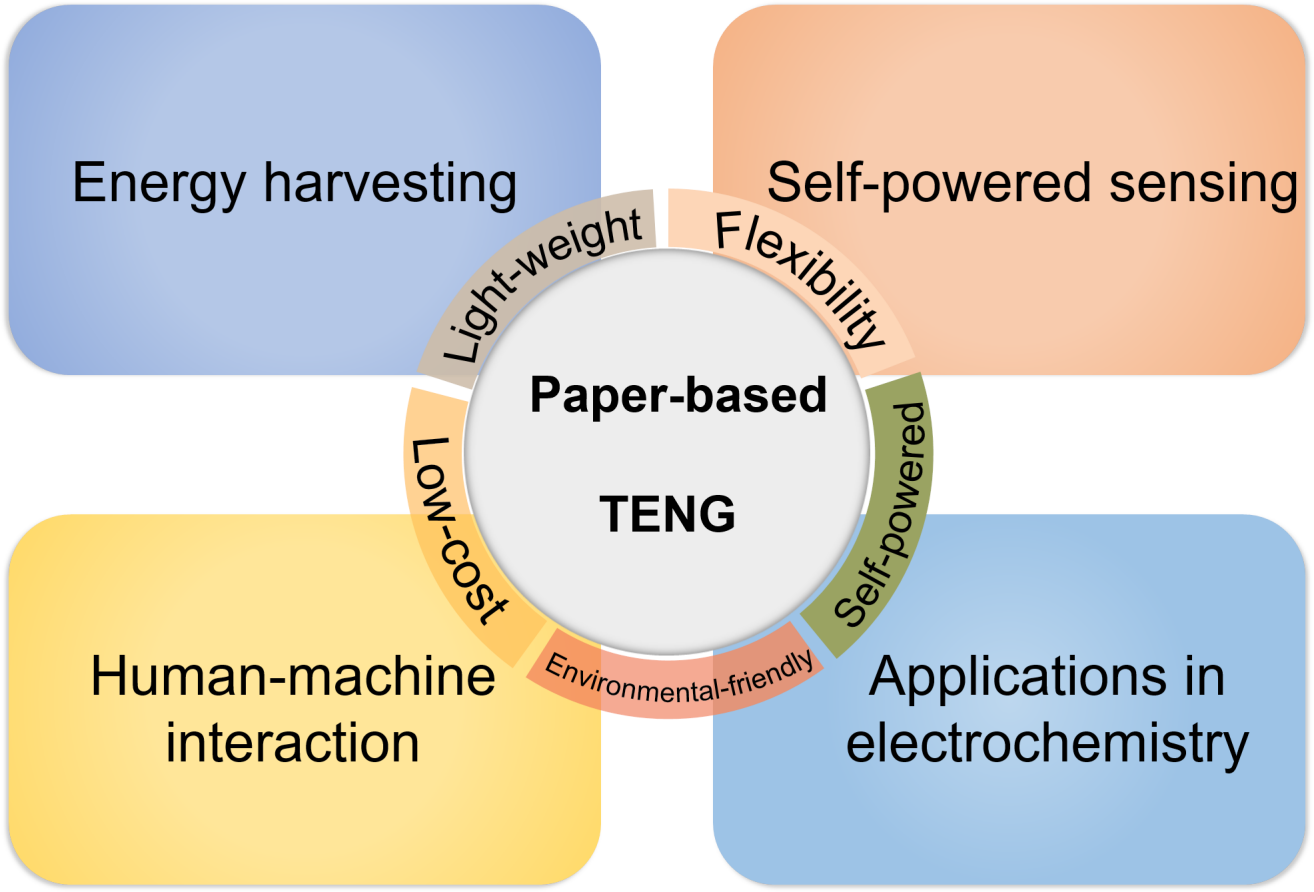
P-TENGs and their applications.

Based on the above advantages and conveniences, paper-based TENGs (P-TENGs) have exhibited great potential for many practical applications, leading to a simple yet effective way for the next generation of energy devices and paper electronics. In this review, we try to look back and summarize the latest developments in the field of P-TENGs. Figure 1 schematically shows the theme of this review article and several typical examples in which P-TENGs are used. This paper starts with an overview of TENGs and the corresponding working mechanism of four basic working modes based on charge transfer and on the electron-cloud potential-well model. Regarding surface modification and fabrication methods involving paper, we then highlight the strategies to improve the output performance of P-TENGs. In another section, we give a detailed review on the application of P-TENGs, with two-dimensional patterns and three-dimensional structures, on self-powered sensing devices, human–machine interaction, electrochemistry and highly efficient energy-harvesting systems. To conclude the review, perspectives and proposals regarding future potential applications and research directions are discussed.

## Review

### Four working modes of TENGs and charge-transfer mechanisms

**Figure 2:**
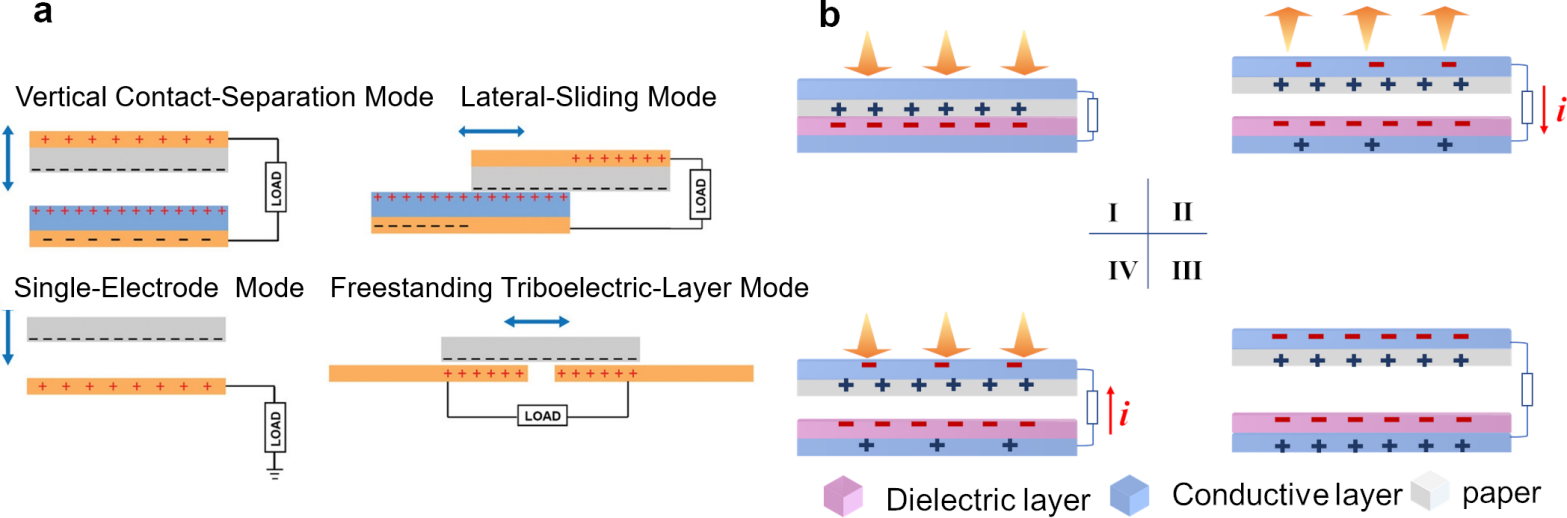
(a) Four working modes of TENGs [87]. Copyright © 2018 WILEY‐VCH Verlag GmbH & Co. KGaA, Weinheim. Adapted with permission from Changsheng Wu et al., “Triboelectric Nanogenerator: A Foundation of the Energy for the New Era”, Advanced Energy Materials, John Wiley and Sons. (b) Working mechanism of a TENG based on the vertical contact–separation mode.

TENGs, which are emerging and efficient apparatus for energy conversion, have been attracting significant attention from the fields of energy harvesting and self-powered systems. The triboelectric effect [83–85], a type of contact-induced electrification, is the basis of TENGs. It was found that when two different materials are in physical contact, their interfaces become electrically charged. Due to contact electrification (CE, or triboelectrification) [86], opposite charges will be induced when the two materials are separated by a mechanical force, which will correspondingly generate a potential difference between the two materials due to electrostatic induction. If an electrical load is connected through an external circuit, the previously induced potential difference will drive the electrons to flow between the two materials (through the electrodes and the external circuit). Depending on the circuit configurations and on the variations in the polarization direction, TENGs can have four working modes [87], including vertical contact–separation (CS) mode, in-plane lateral-sliding (LS) mode, single-electrode (SE) mode, and freestanding triboelectric-layer (FT) mode, as shown in Figure 2a.

In the vertical CS mode, a stack of two dielectric films is plated with a metal electrode at the back surface of each layer. When the two dielectric films are vertically separated and periodically contacted due to the application of external forces, a small air gap is formed in the middle and a potential difference is induced between the two electrodes, which can drive the forward/reverse flow of charges via the external circuit. The in-plane LS mode relies on the relative slippage between the two materials in a horizontal direction parallel to the surface. An alternating current output can be produced during the sliding motions between the top and the bottom layers. This kind of slippage is also common in a variety of rotation-induced sliding modes, which exhibit huge potential for application in high-output TENG devices. Compared with the vertical CS mode and the in-plane LS mode, the SE mode has only one electrode at the bottom, which is connected to ground and taken as the reference electrode. The direction of the induced electric field can be reversely changed during the approximation or separation between the bottom electrode and the upper dielectric materials. The charge exchange will occur between the bottom electrode and ground to balance the induced potential variation. The application scenarios of TENGs with an SE mode are broad, including direct finger/hand/skin touch or body motions. The FT mode uses two unconnected symmetrical electrodes as the reference electrodes. When the top free-standing (i.e., noncontact) dielectric layer moves from one electrode to the other, electrostatic charges will be induced on the two electrodes in sequence. Similar to the SE mode, if one takes one electrode as the reference electrode, the induced charges will flow from the reference electrode to the other electrode through the external load. Thus, the electrical output is induced by the asymmetric charge distribution during the suspending (forward/backward) movements.

Based on the combination effect of triboelectrification and electrostatic induction, the working mechanisms of the four operational modes of TENGs are similar. Taking P-TENGs in the CS mode (most common design in previous works) as a typical representative example, we further systematically analyze the working mechanism of the detailed charge-transfer process. Figure 2b elucidates the charge generation and the electron-transfer process at the friction interfaces (paper/the other dielectric layer) and electrodes (upper/bottom electrode) during one contact–separation cycle of P-TENGs. The electrification occurs at the interfaces between the paper and the other dielectric layer owing to different electronegativities when they come into contact. Different triboelectric charges (positive and negative charges) are induced by the same amount on the surfaces of the friction layers. As there is no electric potential at this stage, there is no electron transfer between the two conductive layers (Figure 2b-I). When the two friction layers start to separate along the vertical direction, opposite charges are induced in the upper and lower conductive electrodes owing to electrostatic induction (Figure 2b-II). As the distance between the two layers increases, the electric potential difference between the two layers enhances, driving the electrons to flow through the external load which generates an instantaneous current. When the two layers are separated by a maximum distance, the positive and negative triboelectric charges become fully equilibrated, resulting in no current flow through the load (Figure 2b-III). When the two layers approach each other, the electrostatic charges are induced and accumulate again, driving the electrons to flow through the load between the two conductive layers in a reverse direction (Figure 2b-IV). Finally, the two friction layers become fully in contact and the whole system returns to the initial state. At this stage, the triboelectric charges are completely balanced and there is no output current.

Although the origin of the contact electrification has been a matter of debate for a long time, no conclusive model to explain this phenomenon has been proposed. Previous studies investigated whether the electron or ion transfer were dominant in the contact-electrification phenomenon. However, the results were highly controversial [88–89]. Xu et al. [90] have proposed that the quantification of the surface charge density at different temperature values is a critical method for investigating this phenomenon. This can be readily explored as an effective tool to identify the transferred charges and the corresponding CE mechanism in TENGs. The results shown in [90] suggest that the electron transfer dominates the CE process. The charge retention ability is attributed to the intrinsic potential barrier heights of the different materials, which can prevent the charge dissipation. As the CE behavior is dependent on the surrounding temperature, Xu et al. [91] have further explored the operation of TENGs at high temperature values. Their results reveal that the thermionic emission of electrons is the main reason for CE and the atomic thermal vibrations strongly influence the CE at increased temperature values.

**Figure 3:**
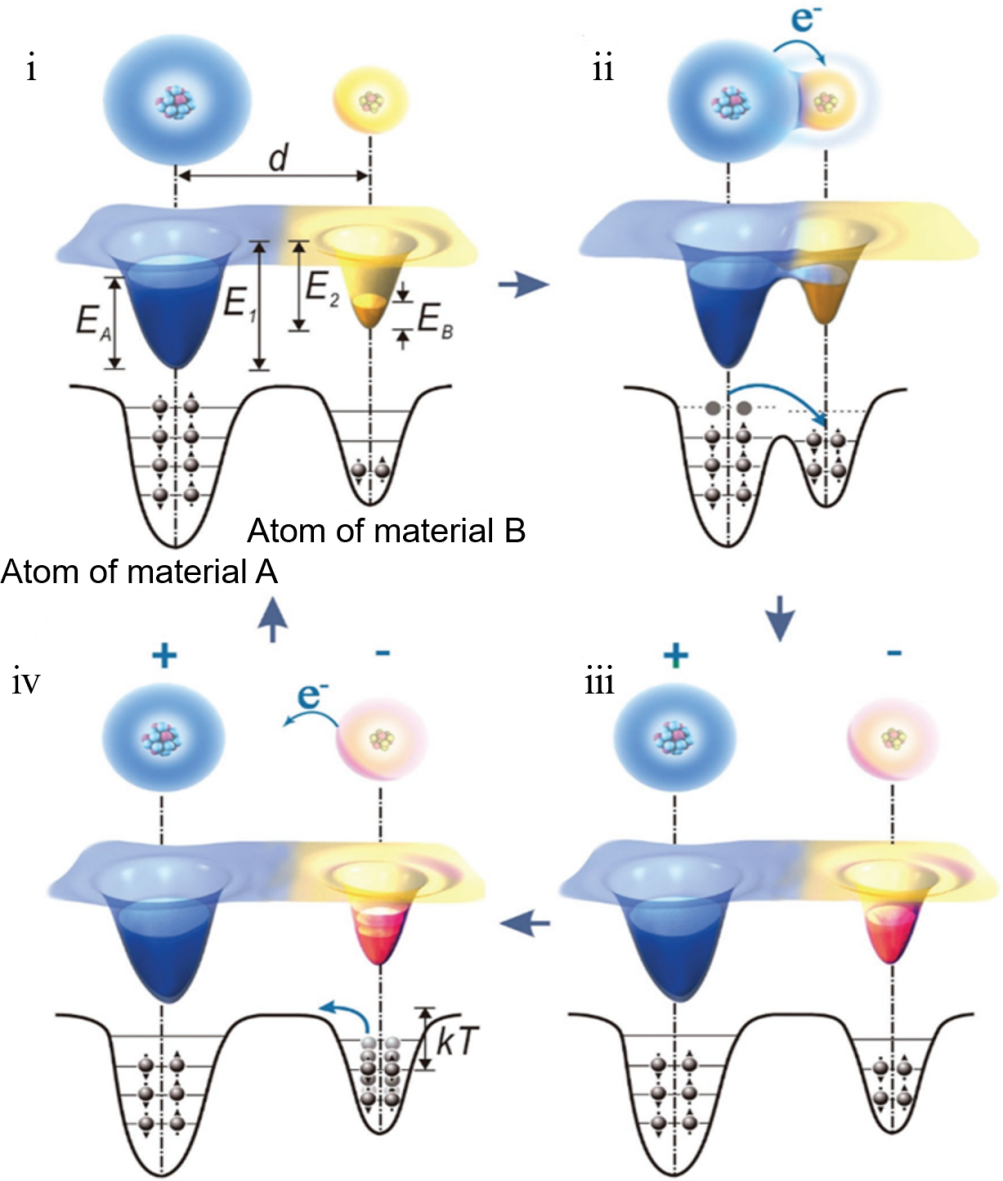
The proposed electron-cloud potential-well model for electron transfer, which is the dominant mechanism of contact electrification [90]. Copyright © 2018 WILEY‐VCH Verlag GmbH & Co. KGaA, Weinheim. Adapted with permission from Cheng Xu et al., “On the Electron‐Transfer Mechanism in the Contact‐Electrification Effect”, Advanced Materials, John Wiley and Sons.

To better demonstrate the electron-transfer mechanism, which is dominant in CE, Xu et al. have proposed an electron-cloud potential-well model (Figure 3). At the initial state, since the highest occupied energy levels of the two materials (A and B) are different and the individual electron clouds of the materials are separated by a distance *d*, the electrons cannot be transferred between material A and material B. The trapping effect of the potential wells prevents the electrons from escaping (Figure 3-i). Once the material A comes into contact with the material B, their electron clouds collide with one another to form ionic or covalent bonds. The overlap between the electron clouds enable the electrons to spontaneously flow from material A to material B (Figure 3-ii). The majority of the transferred electrons will remain in B even if the material A is separated from the material B by an enlarged energy barrier. This leads to A and B being positively and negatively charged, respectively (Figure 3-iii). As the temperature rises, the transferred electrons in B tend to more easily escape from the potential well and to be thermionically emitted into the air, leading to a gradual decay of the surface charges (Figure 3-iv).

### Treatment methods for paper and P-TENGs

The electrical properties of paper are critical determinants of the performance of P-TENGs. The original paper structure usually does not meet all the requirements for the desired applications on P-TENG devices. Therefore, corresponding treatment processes (e.g., deposition of conductive materials by laser patterning, screen printing, spray coating, thermal deposition, surface morphology engineering, and chemical modification [46,92–98]) are often applied to convert paper into a conductive electrode or into a charge-enriched friction layer to improve P-TENG output performances. As the TENG output performance closely depends on the triboelectric polarity of the friction layer, the engineering of friction layers with more charges with an opposite polarity induces a larger triboelectric charge density. Since paper is mainly composed of cellulose, which generally shows a tendency of losing electrons (electropositive), it is preferred to pair paper with a friction material that can easily gain electrons (electronegative) according to the triboelectric series [83,99–100].

**Figure 4:**
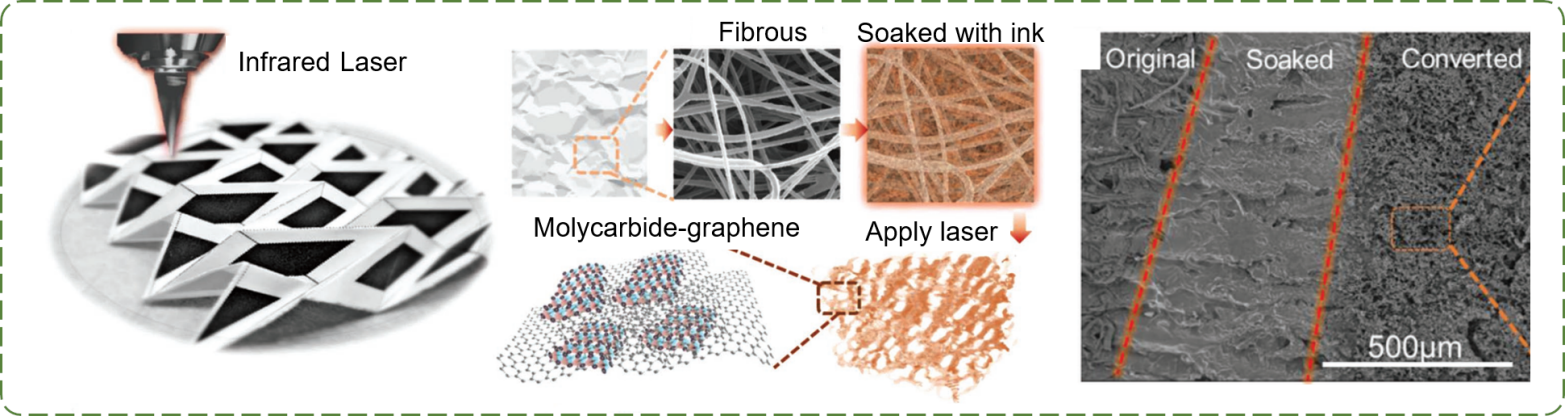
Treatment methods for paper and P-TENGs. Schematic illustration of a simplified MCG composite obtained by a direct laser-writing MCG-patterning process. An SEM image of a paper depicting three regions (from left to right): paper, paper soaked with a Mo–gelatin ink, and paper with converted MCG composites [92]. Copyright © 2018 WILEY‐VCH Verlag GmbH & Co. KGaA, Weinheim. Adapted with permission from Xining Zang et al., “Laser‐Induced Molybdenum Carbide–Graphene Composites for 3D Foldable Paper Electronics”, Advanced Materials, John Wiley and Sons.

Figure 4 depicts a paper-based 3D foldable device submitted to a direct laser patterning method, which can convert ink-soaked paper substrates to multifunctional carbide/graphene (MCG) composites [92]. The composites have shown good conductivity even after repeated mechanical bending and folding tests. Moreover, the laser pattering process results in porous MCG structures (with pore sizes ranging from hundreds of nanometers to several microns), which can be used in various applications, such as mechanical energy harvesting devices, chemical sensors, and electrochemical supercapacitors.

**Figure 5:**
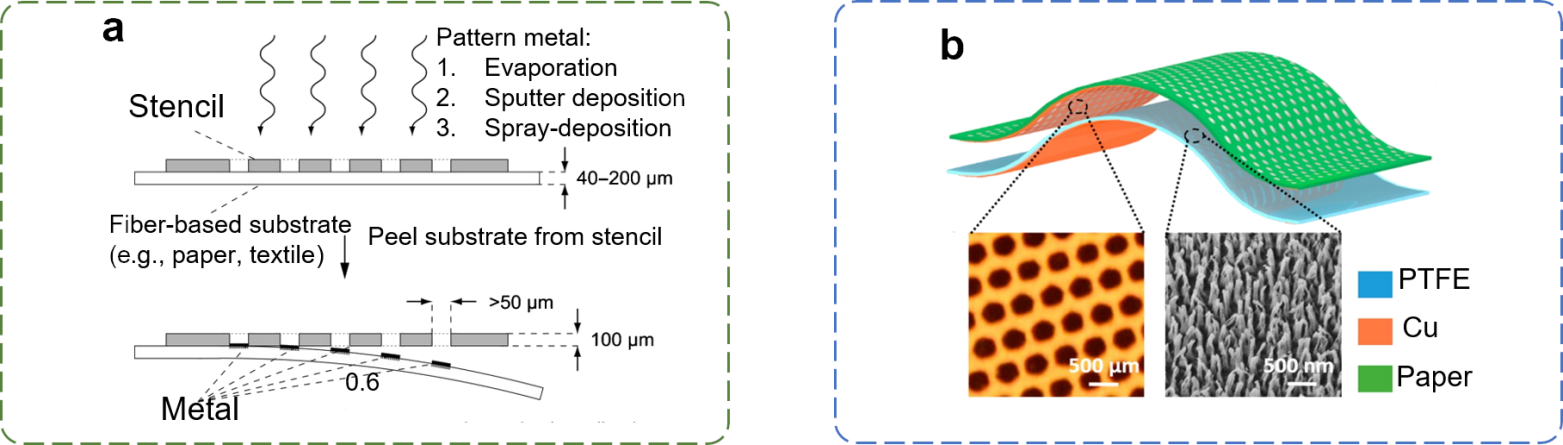
(a) A general scheme for fabricating an electrode on paper substrates [102]. Copyright © 2010 WILEY‐VCH Verlag GmbH & Co. KGaA, Weinheim. Adapted with permission from Adam C. Siegel et al., “Foldable Printed Circuit Boards on Paper Substrates”, Advanced Functional Materials, John Wiley and Sons. (b) An acoustic energy harvester with a multi-hole paper electrode obtained by vapor deposition. Adapted with permission from [103], Copyright 2015 American Chemical Society.

Screen printing is a facile, efficient, high-throughput and low-cost printing method [101]. A layer of ink is scraped across the screen surface and, then, extruded through the open pores of a patterned mesh into the substrate. The printing resolution and the pattern thickness depend on the density of the mesh and on the properties of the ink, respectively. Screen printing has been widely used for fabricating conductive electrodes, semiconducting layers of solar cells, and active materials in field-effect transistors (FETs). It is commonly used as a planar printing technique for batch processing. It is also further adaptable to a roll-to-roll process or to rotary screen printing, which enables a facile and high-throughput printing on curved surfaces. The deposition process assisted with soft stencils is another “bottom-up” method for the preparation of functional materials on flexible and irregular surfaces. Even though P-TENGs require flexible conductive materials, metallic materials (e.g., copper, zinc, silver, and gold) are still frequently used as electrodes for flexible electronics due to their excellent electrical conductivity. By using the Kapton tape to attach soft stencils to paper, various metals can be deposited through the stencils by thermal/e-beam evaporation, sputter deposition, or spray deposition. When the stencils are peeled off they can readily leave the metallic patterns on the paper substrate [102] (Figure 5a). In comparison to the conventional printing techniques, vacuum evaporation and vacuum deposition techniques usually allow a better control over the material thickness, yielding more homogeneous structures. The vacuum evaporation is applicable to a variety of metals at a high rate of up to 50 nm·s^−1^; however, it requires expensive equipment and high-vacuum conditions. The sputtering can be conducted by using less expensive equipment and under lower vacuum conditions; however, the deposition speed is lower than that of the vacuum evaporation. The thickness of the metal layer obtained by e-beam deposition can be up to several micrometers, which is lower than the thickness of the metal layer obtained by sputter deposition (50–300 µm) [39]. In contrast, the spray coating/deposition with stencil is the most inexpensive process and can be applied at room temperature without any specialized equipment. Figure 5b represents an acoustic energy harvester on a paper substrate with multi-hole electrodes obtained by stencil-assisted vapor deposition [103]. The arrays of small holes with various shapes and distributions are drilled on the stencil via laser cutting, and a layer of copper with thickness of 100 nm is deposited onto the electrode via physical vapor deposition. By combining the stencil-assisted deposition and patterning methods it is possible to apply sophisticated electronic devices or e-skins on flexible and arbitrary substrates [75,104–107].

The electronegativity of the friction layer is critical for the triboelectrification properties of TENGs. Surface chemical modification (to introduce electronegative or electropositive functional groups) and micro-/nanostructure engineering (to introduce charge-trapping points to facilitate charge transfer) are effective ways to modulate the electronegativity. The surface modification of the friction materials is performed to implement chemical functionalization by using various molecules [72,93–94,108–113], while surface engineering can be performed by introducing micro-/nanostructured patterns [74,92,114–121] or porous structures [97,122–124]. Feng et al. [93] modified gum wrappers with polydopamine to improve the output voltage of TENGs by approx. 3.5 times in comparison with unmodified TENGs.

Another typical method to prepare nanoscale paper with different electrical properties is by using vacuum filtration (VF) to either obtain dielectric cellulose nanofiber (CNF)- based nanoscale paper or AgNWs-casted conductive paper electrode [125]. The prepared AgNWs/CNF paper is flexible enough to be repeatedly folded and unfolded. When the number of folding/unfolding cycles increases, the sheet resistance of the AgNWs/CNF paper increases only a little, which demonstrates its superior durability and conductivity. VF is a simple and rapid method to cast functional materials onto solution-processable substrates without the need to implement any time-consuming and high-cost processes (e.g., evacuation, thermal/e-beam heating, and radio-frequency sputtering). The conductivity is also easily adjustable by changing the concentration of the conductive precursors (e.g., AgNWs, carbon nanotubes, and reduced graphene oxide).

A special nanoscale paper composed of nanocellulose, which is easy to be synthesized/chemically modified/doped, has attracted great attention in recent years. Generally, cellulose-based composites are used as the electropositive layer, while some common synthetic polymers, such as fluorinated ethylene propylene (FEP), polytetrafluoroethylene (PTFE), and poly(dimethylsiloxane) (PDMS) are used as the electronegative part. As the synthetic polymers have a smooth surface and a dense structure, which are limiting factors for the triboelectric performance of TENG devices, Chen et al. tried to introduce a nitro group into the counter cellulose friction layer to change the triboelectric polarity of cellulose with higher electron-attractive capacity [94]. The hydroxy groups in the cellulose molecules were mostly replaced by nitro groups in order to form a nitrocellulose membrane (NCM), which has a porous structure and a rough surface that improve the triboelectric performance of the constructed TENG. The P-TENGs with a NCM as the negative friction layer have maximum open-circuit voltage (*V*_oc_) and short-circuit current (*I*_sc_) values of 196.8 V and 31.5 μA, respectively. The NCM-based P-TENG also exhibits good stability and durability and can serve as a sustainable power source. More importantly, the utilization of nanocellulose paper offers an inexpensive, renewable, and biodegradable method to fabricate high-performance P-TENGs through a simple and environmentally friendly approach.

### Geometry design of P-TENGs

#### Two-dimensional patterns

Due to its low cost, lightweight, flexibility, and environmentally benign properties, paper has been extensively applied in disposable and flexible electronics. It is also used to generate various structures owing to its relative toughness and rigidity. Two-dimensional patterns can have a variety of different configurations. The Li group has shown ultrasoft, cuttable and inexpensive P-TENGs for harvesting mechanical energy in various forms [126]. The main materials utilized on those P-TENGs were commercial tissue paper and silver nanowires (Ag NWs), which were key to their feasibility. The P-TENGs were built with two conductive paper pieces as the electrodes, which were sandwiched between two commercial tissue paper pieces. Three basic operation modes were proposed for those P-TENGs, considering diverse movement possibilities. These modes consisted of the vertical CS operation mode, the LS mode and the self-contact (SC) operation mode. One of the critical advantages of using P-TENGs is that the vertical CS mode and the LS mode can generate triboelectric charges with any target object materials. In the self-contact operation mode, the P-TENG is folded such that the polyvinyl chloride (PVC) side faces the paper side.

#### Three-dimensional structures: origami

Origami (an ancient paper-folding art) was originated in China with the invention of paper and then refined in Japan. Recently, origami has inspired the design of various devices, ranging from macroscopic structures to microscopic materials. Schenk and Guest systematically described the geometry and kinematics of folded shell and cellular metamaterials [127]. The Zhu group used an instant light-driven walking origami-inspired graphene paper to implement programmed vertical and lateral transformations and to improve the performance of many well-designed motions [128]. By using origami-inspired configurations, Yang et al. demonstrated a sophisticated P-TENG for harvesting mechanical energy from various types of human motions, such as stretching, lifting, and twisting [129]. The origami P-TENG could also serve as a self-powered pressure sensor to distinguish, for example, the weight difference between different coins.

**Figure 6:**
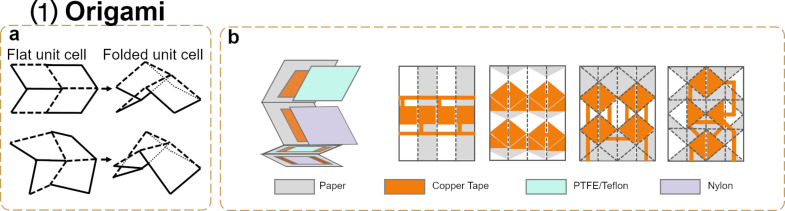
Geometry design of a P-TENG (1) origami: (a) Folded standard (top) and modified (bottom) unit cells. (b) One unit and four different crease pattern designs of SPINs.

Miura-ori, a classic folding structure proposed by Miura, has been applied in the design of certain structures, such as solar sails [130]. Dudte et al. provided a scale-independent optimization algorithm to program the problem of determining generalized Miura-ori tessellations that conform to prescribed surfaces [131]. The flat and folded unit cells of a standard and of a modified Miura-ori structure are depicted in Figure 6a. The complexity of the folding structures is comprised of the basic origami unit cell: four coordinated mountain–valley structures. The basic origami unit cell constitutes the heart of the simplest origami tessellation and can be further engineered into compact and deployable structures of arbitrarily complex geometries. The design of complex origami structures starts with the folding of thin sheets along the creases, which will lead to different geometries in a large scale depending on the thickness and size of the paper sheet. The excellent mechanical properties of the origami structures enable diversified and sophisticated compressions and expansions. Previous research works on paper-based origami mainly focused on the seamless integration of sensing and interactive actuation; however, they lacked to address the concerns regarding paper-based power sources or self-powering strategies. Chen et al. showed the construction of origami P-TENGs as a self-powered paper interface (SPIN) to harvest mechanical energy from contact–separation movements [132] (Figure 6b). The SPIN unit is made of copper tape electrodes, PTFE, and nylon sheet friction layers. The adjustable parameters of the crease patterns, such as shape, size, and orientation, are key to the aesthetics and functionality of the SPIN energy-harvesting units. There are four types of folding structures for an efficient energy harvesting: the stripe fold, the Miura fold, the Yoshimura fold, and the waterbomb fold. The stripe fold is the simplest folding pattern with the maximum displacement in a 1D vertical compression. The Miura fold can be expanded and compressed along multiple axes but it has only one axis of movement due to a negative Poisson ratio. This fold can be compressed in a layered trapezoidal shape along two directions. The Yoshimura fold has a 3D transformation in a spring-like form, which is promising to be explored in different applications. The waterbomb fold is also a basic 2D compression fold used to create unique geometries; yet they are difficult to apply pressure on. The highest *V*_oc_ value of TENGs is obtained in the stripe fold, followed by decreasing *V*_oc_ values obtained in the waterbomb, Miura and Yoshimura folds, in this order. As the surface area of each fold is the same, the main reason for the different outputs is attributed to the differences in the maximum separation distance parameter. Since the power density is defined as the power divided by the contact area between two friction layers, the design editor can be used to estimate the potential power generation by the folding structures according to Ohm’s law.

#### Three-dimensional structures: kirigami

**Figure 7:**
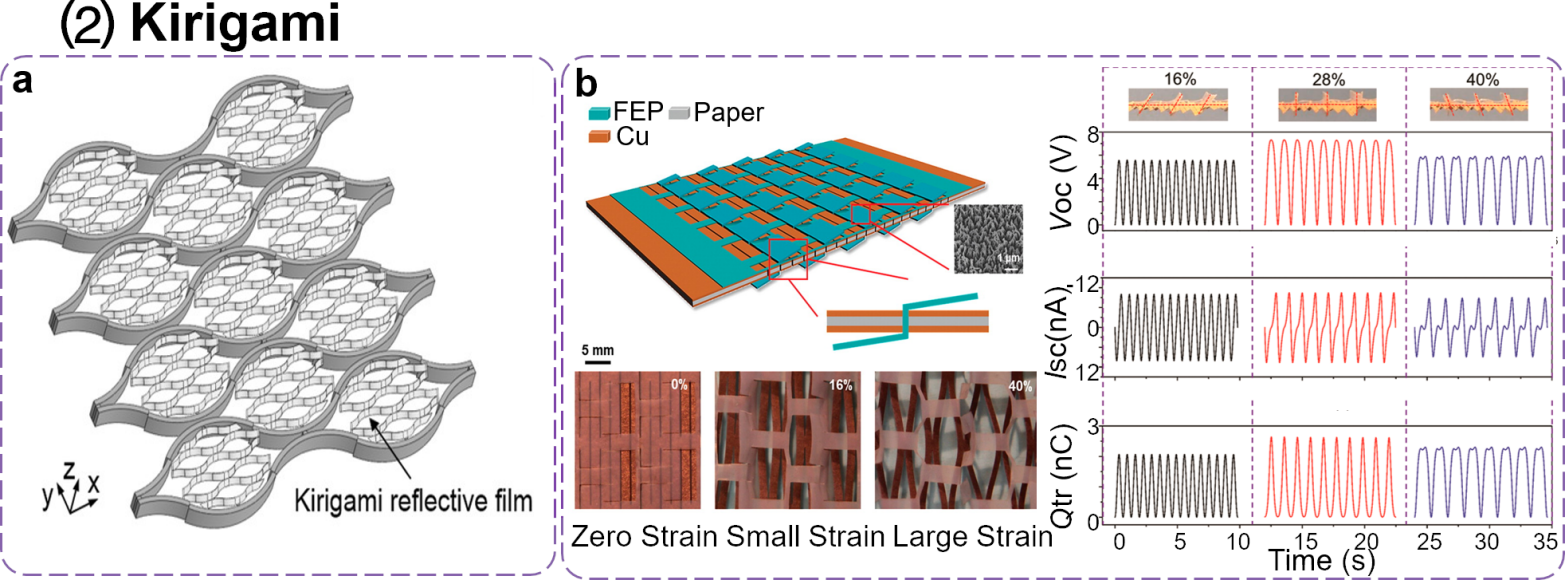
Geometry design of P-TENGs. (2) Kirigami. (a) The profile of the kirigami reflector [133]. Copyright © 2017 WILEY‐VCH Verlag GmbH & Co. KGaA, Weinheim. Adapted with permission from Wei Wang et al., “Kirigami/Origami‐Based Soft Deployable Reflector for Optical Beam Steering”, Advanced Functional Materials, John Wiley and Sons. (b) An schematic structure and photographs of the different tensile strains of K-TENGs and the typical electrical outputs of K-TENGs. Adapted with permission from [136], Copyright © 2016, American Chemical Society.

Kirigami is a combination of folding and cutting; therefore, it is different than origami, which only involves the folding of thin planar sheets. The introduction of additional cuts can further enhance the deformability of the resulting structures. Inspired by the art of paper cutting, Wei Wang et al. designed a soft deployable reflector for optical beam steering using kirigami [133]. As shown in Figure 7a, the planar soft deployable structure is made of uniform hollow pockets in which the kirigami reflective films are inlaid. As the axial strain is increased during stretching, the surfaces of the kirigami reflector become parallel. By changing the characteristic angle, the deformation angle of the reflective film and the slit patterning can be controlled.

The kirigami design in TENGs is a highly promising strategy for energy harvesting due to the introduction of more contact/separation spaces. Guo et al. reported a cut-paper-based self-charging power unit (PC-SCPU) by combining TENGs and the SC mode, which is capable of simultaneously harvest and store energy from body movements [39]. Zhou et al. also proposed an all-in-one stretchable self-charging power unit based on folded carbon paper TENGs operating in SC mode [134]. Xia et al. proposed a milk-based P-TENG (MP-TENG) to increase the output performance of TENGs [114]. A lozenge-stacked MP-TENG was designed and fabricated with an enhanced output. Zhang et al. proposed a rhombic-shaped paper-based triboelectric nanogenerator (RP-TENG) that can effectively operate under tensile strain conditions and with an applied contact force [135]. The Wang group developed a highly stretchable interlocking kirigami TENG (K-TENG) based on inelastic paper materials [136]. K-TENGS are capable of harvesting mechanical energy from various types of motions, such as stretching, pressing, and twisting due to the thin cutting film design. The schematic structure of K-TENGs and related photographs are illustrated in Figure 7b. The prepared FEP film with a rectangular kirigami pattern was treated by inductively coupled plasma, in which the parameters of a unit cell were designed to match those of the linear kirigami on paper. The copper-coated paper was chosen as one of the friction layers and as the electrode for the K-TENG, whose horizontal and vertical spacing between the notches was set as 2 mm. The resulting K-TENGs could withstand a tensile strain of up to 200% without breaking. The typical electrical output of a K-TENG under cyclic stretching of up to specific tensile strain values of 16%, 28%, and 40% were tested. When the tensile strain of a given K-TENG was at 16% or 28%, the *V*_oc_ and the transfered charge (*Q*_tr_) increased monotonically with the applied strain, while the *I*_sc_ only changed direction at the maximum applied strain and at zero strain. When the tensile strain of a K-TENG was at 40%, *V*_oc_ and *Q*_tr_ first increased with the applied strain until they reached a fully stretched state, after which both values started to decrease.

### Emerging applications of P-TENGs

#### 2D and 3D self-powered sensors based on paper-based TENGs

As a promising technology for self-powered sensing devices, TENGs can directly convert mechanical stimuli into electrical signals without additional transducers. Therefore, TENGs have a great application potential in the fields of active-sensing devices and self-powered sensors [137–144], which require less standby power consumption and simpler control circuits compared to traditional sensors. More recently, P-TENGs have also been employed in a wide range of application scenarios, including acoustic [103], pressure/force/weight [129,145–146], velocity/acceleration [136,147], position [148], anti-theft [149], and temperature [145] sensors. Liu et al. [146] reported a self-powered active P-TENG force sensor with an ionogel-infiltrated paper (IIP) as the electrode, aiming for a flexible all paper-based sensor. The upper ionogel-infiltrated paper-based flexible electrode was adhered to the back side of the filter paper as the upper triboelectrification layer, while the counter friction layer corresponded to the bottom ionogel-infiltrated paper attached inside of the filter paper. The bottom ionogel-infiltrated paper was in contact with the upper pristine filter paper to generate the induced electrostatic charge. In addition, it worked as an electrode conducting the electrons to the external circuit. The all paper-based flexible sensor could detect different impulsive forces without any extra power consumption.

Based on efficient conversion of ambient mechanical energy into electricity, P-TENGs have been extensively utilized in self-powered mechanosensing devices [150] (e.g., pressure, tactile, strain, and force sensors). Some special but also frequently required applications (e.g., humidity and height sensors) have been rarely reported. Recently, Ejehi et al. proposed a self-powered humidity sensor based on a graphene oxide (GO) paper-based TENG [151], which showed an outstanding power density as high as 1.3 W·m^−2^, a *V*_oc_ of up to 870 V and a *I*_sc_ of 1.4 µA·cm^−2^. GO was chosen to be precipitated on paper in virtue of the reactive oxygen functional groups and due to its high specific surface, which made it a good candidate for both energy harvesting and material storage [152]. Moreover, its intrinsically high mechanical strength enabled the production of a flexible and stable thin layer, which could be used as a free-standing friction layer (also electrode) in TENGs.

**Figure 8:**
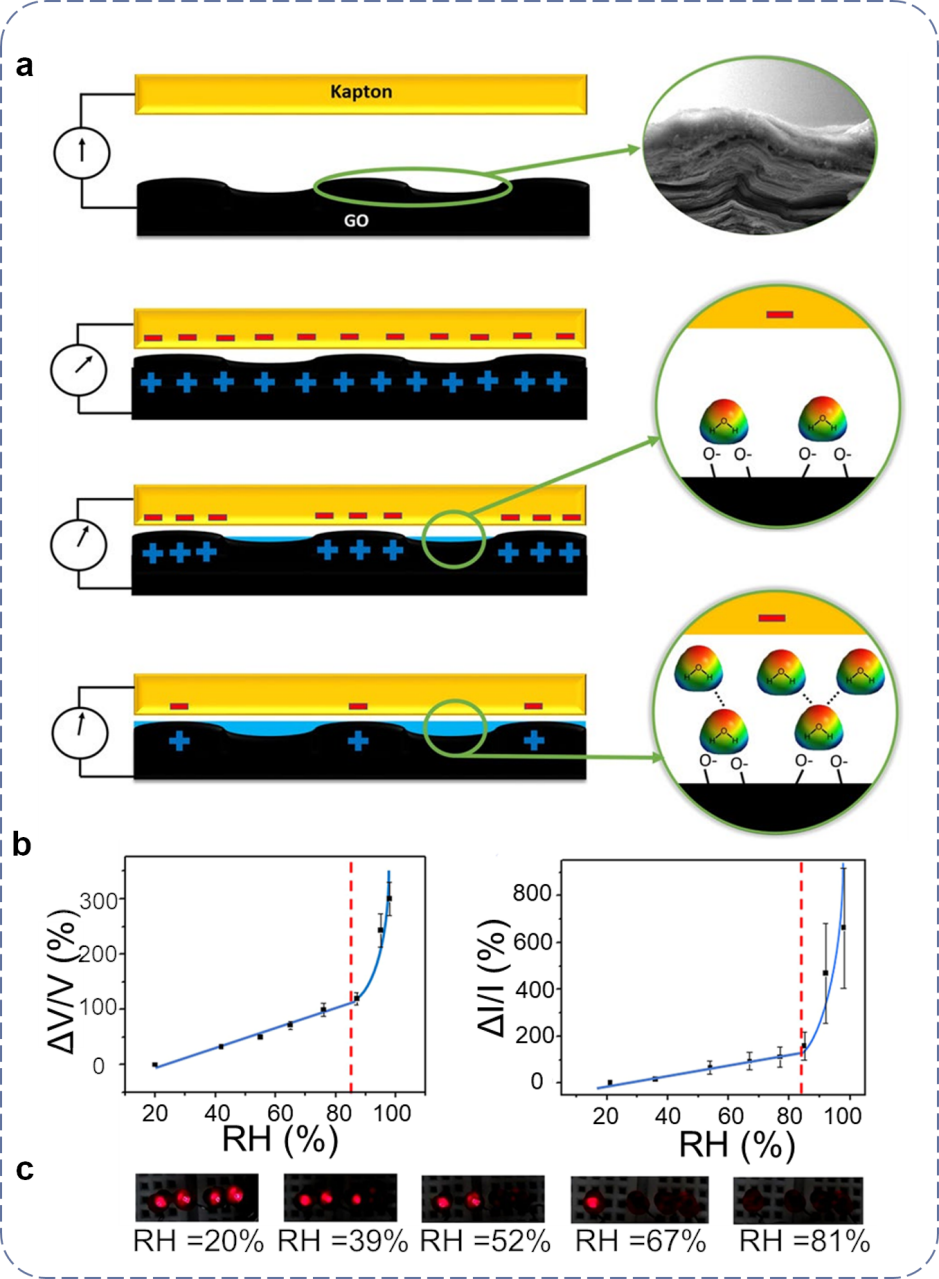
3D self-powered sensors based on P-TENGs. (a) The sensing mechanism of the self-powered GO paper-based TENG humidity sensor. (b) The response value of the electrical output as a function of RH. (c) The number of LEDs which can be lit by a GO paper-based TENG at different values of RH. Figure 8 is adapted from [151] (© 2020 Faezeh Ejehi et al., published by Springer Nature; distributed under the terms of the Creative Commons Attribution 4.0 International License, https://creativecommons.org/licenses/by/4.0).

In addition to the applications in energy harvesting, GO can also be readily used to monitor the relative humidity (RH) because of the strong interactions between water molecules and oxygen functional groups on its surface. The basis of a GO humidity sensor is the variation of impedance or capacitance owing to the tendency of the water molecules to be adsorbed on the GO surface. The rich functional groups of GO and the flexibility of GO paper deliver the significant potential for a less complicated TENG structure with a high sensitivity and a wide range of humidity detection. The corresponding mechanism of a self-powered GO P-TENG humidity sensor is displayed in Figure 8a. In general, GO P-TENG sensors respond to humidity due to the physical adsorption of water molecules on the surface of the GO layer. In a low RH scenario, water molecules are primarily condensed onto the available active sites of the GO surface, which act as the obstacle for electrostatically induced charges and reduce the contact surface to form a depletion region. As a consequence, the output current of the GO P-TENGs decreases in a low RH condition. As the RH increases, the hydroxy groups of the first physisorbed layer bond to water molecules, through hydrogen bonding, which also permeate into the interlayers of GO. The gradually absorbed water layer creates a uniform barrier layer for the induction of positive charges on the GO surface, which results in the formation of a continuous depletion region and leads to a more rapid current decrease at a higher RH. In order to investigate the electrical performances of the fabricated GO P-TENG as a self-powered humidity sensor, the normalized *V*_oc_ and *I*_sc_ of the GO P-TENG were tested, both of which showed an elevated slope when the RH was increased. The response value of the generated voltage reached 400%, while the maximum value for the current response was approx. 700% at a RH = 99% (Figure 8b). To illustrate a more vivid demonstration, the number of LEDs powered by the GO P-TENG decreased with a gradually increasing RH, as shown in Figure 8c. Compared with previously reported TENG-based humidity sensors, in which a TENG is generally used as the external source to power the humidity sensor, the GO P-TENG reviewed here is the first self-powered humidity sensor that utilizes the output performances as sensing signals. The achieved high sensitivity of 500% (*V*/*V*-%RH) is useful for portable/wearable electronics and for potential industrial applications due to its flexibility and lightweight.

Regarding height sensors, Xia et al. proposed a stacked 3D zigzag-structured P-TENG [70], which displayed different output signals depending on the changes in height of a falling object or on the impact when this object came into contact with the P-TENG. To increase the overall output, the paper in the zigzag-structured P-TENG was coated with commercial conductive ink. The ink-coated paper worked as both the functional friction layer and as a supporting structure, while PTFE was used as the other friction layer. The detailed working process is described as follows: at the initial state, a bouncy ball was placed at a certain height. At that stage, the surfaces of PTFE and of the paper of the height sensor were completely separated. When the TENG sensor was compressed by the impact of the falling bouncy ball, the induced triboelectric output could be detected. With the bouncy ball bouncing back up, the PTFE and the supported conductive paper began to separate.

Xia et al. designed a sliding triboelectric nanogenerator based on paper (SP-TENG) for self-powered speed and force sensors [147]. In the initial position, the surfaces of the paper and Teflon tape were completely overlapped and in touch with each other. Because of the triboelectrification, the surfaces of the paper had a positive charge, and the surfaces of the Teflon tape had an equivalent negative charge. When the positively charged top plate started to slide outwards, an induced current was generated. When the top plate started to slide in the opposite direction, the induced current flowed back with the help of external loads in order to maintain the electrostatic equilibrium. When the two plates reached the original position, the charged surfaces went back to a complete interaction setting. The SP-TENG can be used as a force and as a velocity sensor. The output voltage of the SP-TENG is approximately linearly proportional to the velocity.

#### 2D and 3D human–machine interaction devices based on P-TENGs

The rapid development of IoTs and of the 5th generation communication technology aims to increase information exchange and communication through the internet. This will require millions or trillions of sensing nodes and systems to enable a convenient, safe, and sophisticated interaction between humans and electronic devices [153]. Although the power required for the operation of each sensor is down to the microwatt level, the number of these units is usually huge. The most conventional technology for powering IoTs (e.g., traditional chemical batteries) faces a significant challenge due to a limited lifetime and powering distance, and due to the wide demand from the broadly distributed sensor networks. It is, therefore, urgent to investigate self-powered devices in order to promote the development of IoTs. TENG is considered a promising candidate for a self-powered mechanosensing device and it has been successfully employed in human–machine interaction. He et al. developed a wireless human–machine interaction system for document management and a smart system for calculation and reading based on a P-TENG with 2D patterns [154]. A wireless sensing system was also developed by integrating a P-TENG in a single-electrode mode, in which the generated electrical signals were direct responses to external mechanical stimuli. The wireless sensing system could be easily operated by the output voltage generated by the touch between finger and paper, which effectively triggered the processing circuit to produce an infrared (IR) signal. This remote IR signal could be detected by a wireless receiver and read by a computer. When the document was moved, a voltage output was generated due to the separation action, which triggered the remote controller and the IR sensor. Through a signal processor, the relative document number was displayed on the monitor, achieving the document management system.

Another application in human–machine interaction is the paper-based calculator, which was fabricated by using 16 separated squared TENGs as functional keys. It was observed that the corresponding numbers and operative symbols were entered and displayed on the monitor when the relevant functional keys were touched by finger. Notably, the effective working distance to trigger the remote-control circuit was within six meters. The wireless sensing system used a self-powered P-TENG that combines the ultrathin and lightweight features of paper with additional advantages, such as cost-effectiveness and versatility, in applications in which the signal triggered by mechanical motions can be converted into a control signal and readily employed in human–machine interactions.

P-TENGs can also work as tactile sensors to obtain a self-powered paper piano controlled in real time. By properly cutting and folding, a paper piano was obtained by utilizing an array of P-TENGs as an interactive keyboard [94]. In the paper piano, a printed paper substrate with a back Cu electrode was used as one of the friction layers, while a nitrocellulose membrane was used as the other friction layers in the P-TENG device. The P-TENG-based keyboard paired with bridge rectifiers and capacitors was connected to a laptop through a microcontroller. By pressing and releasing the keys of a P-TENG-based keyboard, the capacitors were charged and the voltage of the capacitor connected to the key submitted to press–release cycles was measured. The voltage changes of the capacitor were measured as input signals and recorded by a microcontroller, which could trigger the microcontroller and play music in the computer through the embedded program.

**Figure 9:**
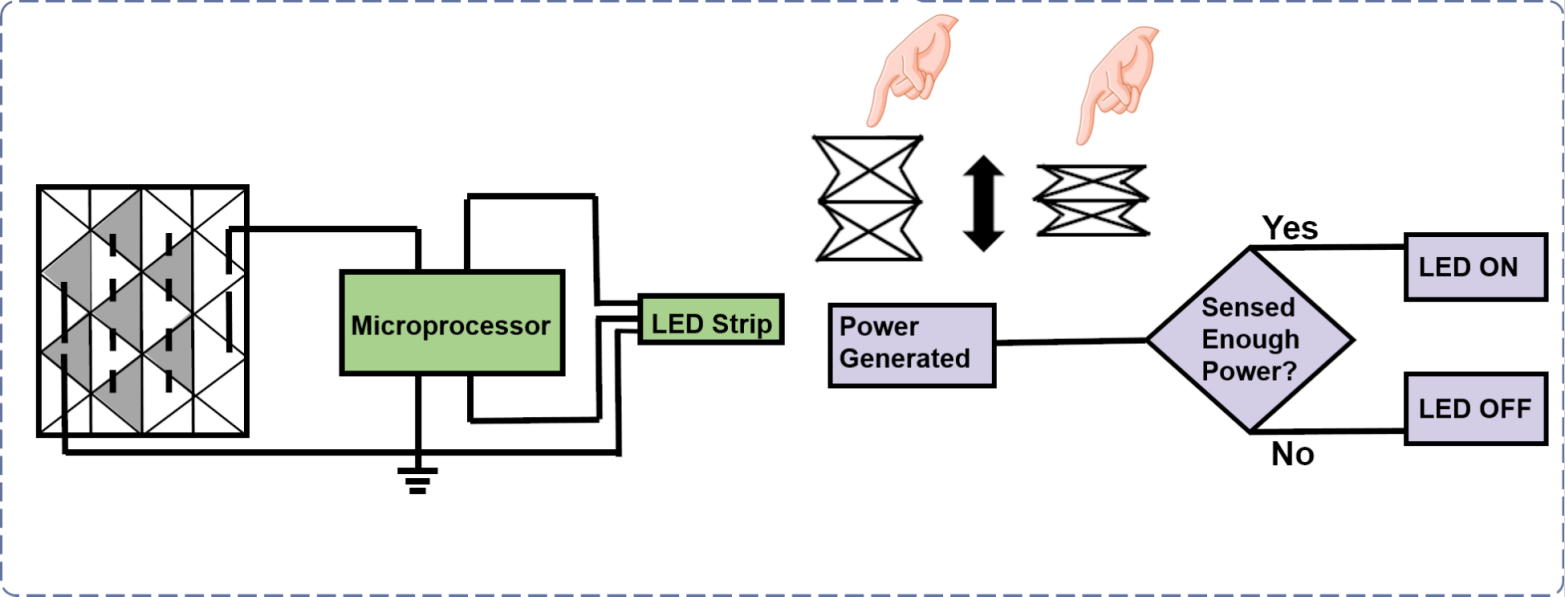
Human–machine interactions based on a 3D P-TENG. Schematic showing a logic flowchart of the Yoshimura fold being used as a voting button in a sensing device.

Chen et al. employed a 3D-structured P-TENG and took advantage of the paper creases to implement repetitive push-and-pull movements in promising applications, such as self-powered mechanosensing systems, electronic skins, and human–robot interactions [132]. In that work, a vote-counting button based on the Yoshimura fold was employed as a force and touch sensing device, as shown in Figure 9. This application adapted the nature of the Yoshimura fold, which provides a spring-like feature that can be pressed and recover automatically. By tapping the “voting button”, which was associated with other relevant items, a triboelectric voltage was generated and recognized by the microprocessor in order to evaluate the induced voltage values and to light a corresponding number of LEDs in a strip.

#### Applications of 2D P-TENGs in self-powered electrochemistry

Metal corrosion is a common phenomenon and a considerable amount of resources are necessary in order to prevent it. Therefore, the development of technologies to protect metals against corrosion has a significant economic importance and has attracted attention from different sectors. Cathodic protection is an effective and a traditional method to keep metals from corrosion. It can be obtained by an external direct current or a by a passive sacrificial anode [155–157]. However, traditional cathodic protection needs external electricity, which is very complex and energy consuming. Assisted with a self-powered system, an ideal cathodic protection system is expected to be sustainably powered by using nanogenerators. This can effectively decrease the usage of traditional methods which are energy consuming and introduce serious environmental problems. Some examples of self-powered systems have been introduced to solve this problem by integrating P-TENGs into an anti-corrosion protection system. Feng et al. have reported P-TENGs that can readily convert kinetic energy into electricity to protect A3 steel from corrosion and biofouling [93]. A square-shaped A3 steel piece was connected to the cathode and a carbon electrode was connected to the anode, with a P-TENG paired with a rectifier and a capacitor connected in parallel with the electrochemical system. The P-TENG, in this case, was composed of PVDF and paper as the friction layers. The maximum values of *V*_oc_ and *I*_sc_ of the P-TENG were 1000 V and 30 mA, respectively. By converting the output form of the alternating current into a direct current with the rectifier, it was possible to efficiently protect the iron electrode from corrosion through the cathodic protection system. The comparative results shown in the photographs of the A3 steel pieces with and without a self-powered cathodic protection system revealed that there were no obvious changes in the morphology and color of the surface of the A3 steel in connection with the TENG. However, the A3 steel without the self-powered protection component was severely corroded. The Tafel plots of A3 steel with and without a cathodic protection showed that with a self-powered cathodic protection provided by the TENG, the A3 steel showed a much lower charge-transfer resistance value and the corresponding Nyquist plot exhibited an arc with a smaller value, which indicated that the TENG-powered cathodic protection system has obvious anticorrosion properties.

Marine fouling, caused by metal contamination and accumulation of biological organisms, is a major challenge for the oceanic ecosystem. With the growing threat to the environment caused by pollution, the search for innovative, highly efficient and cost-effective approaches to curb pollution has attracted much attention [158]. Previous research works have proven that a pulsed electric field can prevent biofouling from adhering to material surfaces [159–161]. Two types of marine algae (*Dunaliella* and *Navicula*) were used as typical indicators to simulate the ecological environment of the marine fouling organisms. Two stainless steel pieces were connected to the anode and to the cathode with a rectifier, and another piece of stainless steel was directly immersed into the algae medium as the blank sample. After introducing the TENG-powered antifouling system, the stainless steel pieces on both the anode and cathode showed good antifouling properties, as indicated by algae density.

**Figure 10:**
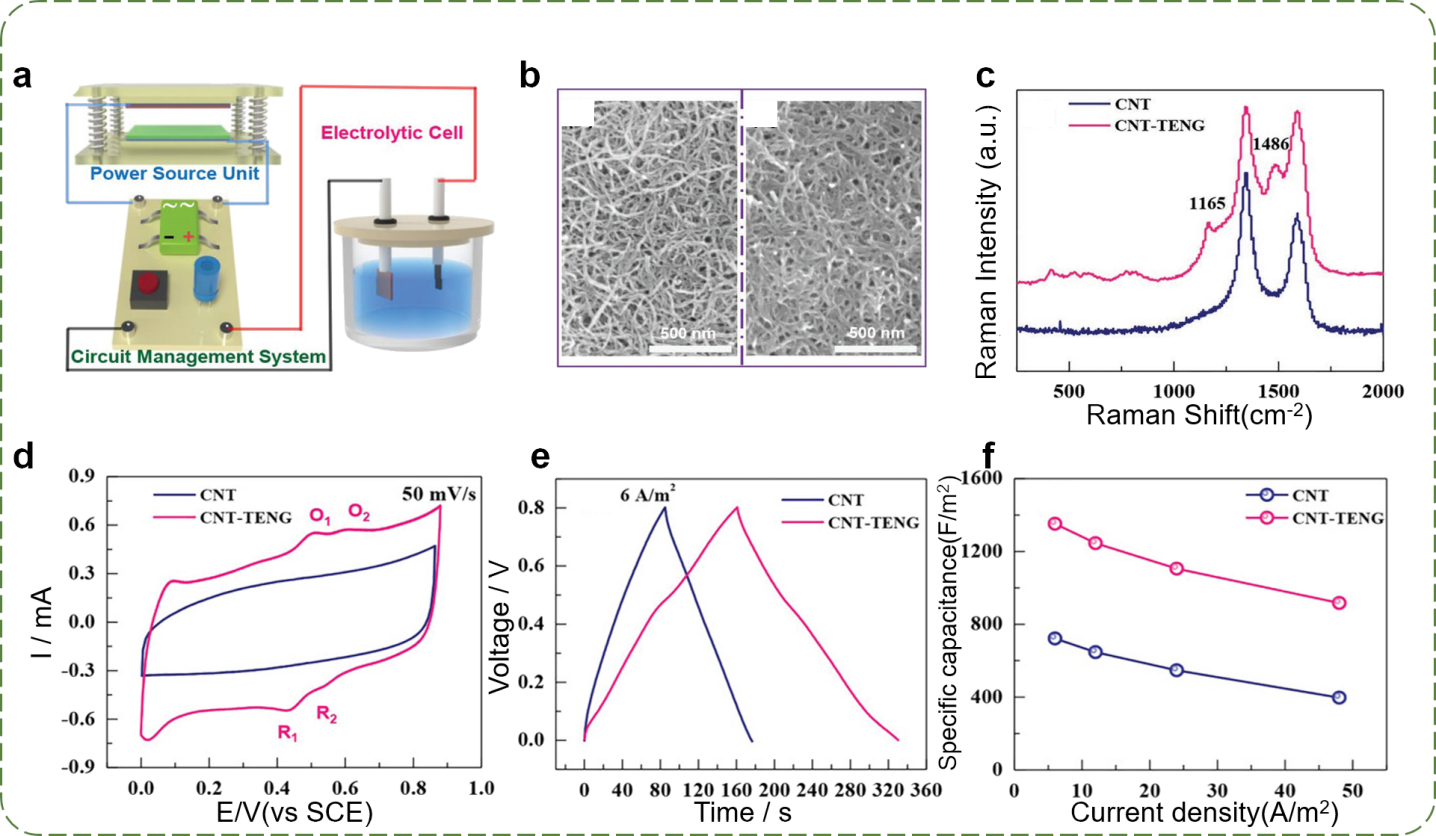
Applications of 2D P-TENGs in a self-powered electrochemical system. (a) Schematic illustration of a TENG electrochemical system and its electrochemical characterization. (b) SEM images of a pristine CNT and of PANI–CNT surfaces. (c) Raman spectroscopy, (d) CV curves, (e) GCD curves, and (f) specific capacitance values of CNT and PANI–CNT electrodes [162]. Copyright © 2019 WILEY‐VCH Verlag GmbH & Co. KGaA, Weinheim. Adapted with permission from Kunming Shi et al., “Dielectric Modulated Cellulose Paper/PDMS‐Based Triboelectric Nanogenerators for Wireless Transmission and Electropolymerization Applications”, Advanced Functional Materials, John Wiley and Sons.

An electrochemical reaction is an electron-transferring or flowing process between an electrode and a substance. TENGs can also be used to harvest mechanical energy and drive electropolymerization processes without an external power source to drive the electrochemical reaction. Shi et al. obtained a P-TENG electrochemical system for electropolymerizing polyaniline (PANI) on a CNT electrode. Figure 10a shows the schematic illustration of a TENG electrochemical system (including a TENG, a rectifier and a capacitor), which was used to improve the capacitance of the CNT electrode by PANI electropolymerization [162]. The TENG powering component was comprised of a cellulose/BaTiO_3_ aerogel paper as the positive friction layer and PDMS as the negative friction layer. The modified CNTs embedded into a thin layer of PANI by the TENG electrochemical system showed a larger diameter compared with the pristine CNTs, as shown in Figure 10b. The electropolymerization of PANI on a CNT electrode is also identified by the Raman spectra in Figure 10c. The electrochemical behavior of CNT and PANI–CNT electrodes were evaluated by using cyclic voltammetry (CV) and galvanostatic charge–discharge characterization (GCD). Compared with the pristine CNT, two pairs of redox peaks appeared in the CV curve of PANI–CNT electrodes at a scan rate of 50 mV·s^−1^ (Figure 10d). The charge–discharge time of the PANI–CNT electrode was almost two times longer than that of the CNT electrode for one cycle (Figure 10e). The PANI–CNT electrode exhibited a larger specific capacitance (approx. 1400 F·m^−2^ under 6 A·m^−2^) in comparison to the CNT electrode under all the applied current densities (Figure 10f). This application of P-TENGs provides a feasible and an effective way to construct self-powered electrochemical systems with a high output performance, enables the application of TENGs in the preparation of electrodes for supercapacitors with high specific capacitance values, and it is promising for large-scale applications in the environmental sciences.

#### 2D and 3D P-TENGs for energy harvesting

Due to its superior performance (i.e., lightweight, low cost, and several material and structural possibilities for their manufacture) P-TENGs have been broadly used as microscale power sources for self-powered systems by harvesting human motion or ambient energy, such as walking, machine vibration, and wave energy. Harvesting low-frequency energy using P-TENGs is of great importance. They have been as used as an acoustic energy harvester and in the field of portable electronics since the beginning of their development. Both 2D patterns and 3D structures based on P-TENGs can be used for energy harvesting and these designed structures are often combined together rather than being mutually exclusive. The electric output performance (i.e., *I*_sc_, *V*_oc_, *Q*_tr_ and power density) is virtually important and it is the major figure of merit for energy harvesting. The electric output performance of P-TENGs for energy harvesting and functionalities and a comparison between P-TENGs and polymer-based TENGs are summarized in Table 1 and Table 2, respectively.

**Table 1:** Summary of output power values for energy harvesting purposes and functionalities in which P-TENGs are applied.

Ref.	Geometry design	Application	

		Electric output (*V*_oc_, *I*_sc_, *Q*_tr_, power density (mW·m^−2^))	Functionality

[49]	2D	5.3 × 10^5^	
[94]	2D	1.61 × 10^4^	self-powered paper piano
[163]	3D (origami)	1.61 × 10^4^	
[147]	2D	1.84 × 10^4^	
[113]	2D	1800	
[68]	2D	906	active sensor
[125]	2D	693	
[46]	2D	398	self-powered sensing system
[162]	2D	352.5	wireless transmission & electropolymerization applications
[154]	2D	285.6	self-powered sensing system
[103]	2D	121	
[80]	2D	72.5	
[82]	2D	64	
[134]	3D (origami)	32.2	
[96]	3D (origami)	29.5	
[147]	3D (origami)	0.583	
[163]	3D (origami)	0.542	
[129]	3D (origami)		pressure sensor
[149]	2D		page mark and anti-theft sensor
[126]	2D		action sensor
[145]	2D		temperature & weight sensor
[164]	3D (origami)		height sensor
[146]	2D		active and passive mode sensor
[151]	2D		humidity sensor
[136]	3D (origami)		acceleration & book state sensor
[93]	2D		anticorrosion & antifouling

**Table 2:** A performance comparison between P-TENGs and traditional polymer-based TENGs.

	Materials	Typical performance				Ref.

		*I*_sc_	*V*_oc_	*Q*_tr_	Power	

traditional polymer-based TENGs	FEP, metal	120 µA	281–560 V	670 nC	7.96 mW	[54–56]
	PTFE, metal	25.97 µA – 1.32 mA	300–400 V	1000 nC	2.84–3 W	[35,53]
	PDMS, CNTs	180 nA	60 V			[81]
	PDMS, liquid metal nanoparticles	12.06 µA	268 V	103.95 nC	0.32 mW	[67]

paper as an electrode (Au Ag Cu Graphite Carbon)	FEP	30 µA	90 V	75 nC·cm^−3^	–	[39]
	PTFE	2–17 µA	20–110 V	38.4–550 nC	121–906 mW·m^−2^	[47,69,104,130]
	PDMS	18.6 µA	300 V	79.3 µC·m^−2^	32.2 mW·m^−2^	[134]

paper as a friction layer	PDMS & BaTiO_3_ cellulose paper	8.3 µA	88 V	–	352.5 mW·m^−2^	[162]
	crepe cellulose & nitrocellulose membrane	31.5 µA	196.8 V	–	16.1 mW·m^−2^	[94]
	PVDF & polydopamine modified paper	30 µA	100 V	76 µC·m^−2^	–	[92]
	PTFE & BaTiO_3_ cellulose paper	forward-poled BC-TENG *I*_sc_ ≈ 9.8 µA, *V*_oc_ ≈ 170 V reverse-poled BC-TENG *I*_sc_ ≈ 1.6 µA, *V*_oc_ ≈ 44 V				[113]

Due to the advantages of cellulose (i.e., a low cost, lightweight, abundant, and accessible material) cellulose-based materials have also been intensively used, in recent years, in the manufacture of energy-harvesting devices, such as TENGs, (as a positive friction layer or as a direct substrate). P-TENGs exhibiting good stability and durability can be used as a sustainable source to power various portable electronic devices. For example, P-TENGs are able to light up 240 LEDs connected in series through a commercial rectifier bridge. To power relatively high-power electronics, the energy harvested by TENG is generally stored in energy storage devices first. This can be performed by using a bridge rectifier and different types of capacitors. The charged capacitor is then used to power the target electronic devices, such as segmented LED display, liquid crystal display (LCD), and electroluminescence (EL) display units. The cellulose-based P-TENGs have the following electric output performance parameter values: *V*_oc_ (approx. 196.8 V), *I*_sc_ (approx. 31.5 μA), and power density of 16.1 W·m^−2^ [94].

**Figure 11:**
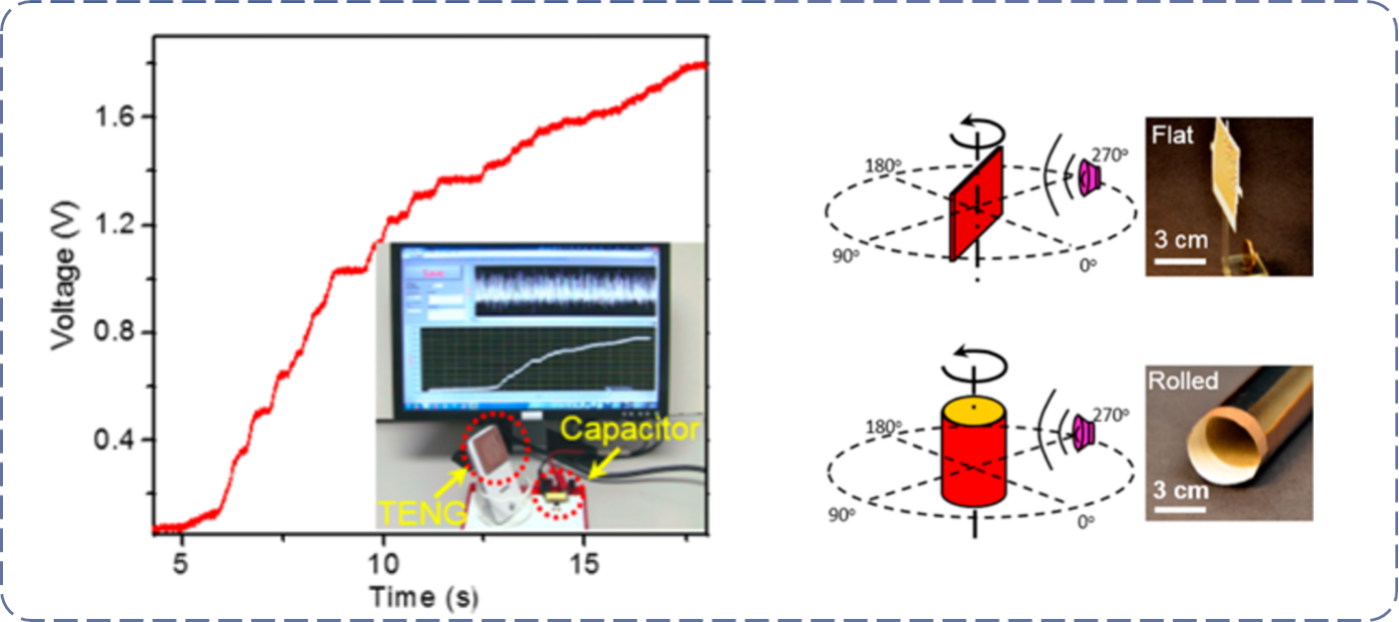
Sound wave energy harvesting by an ultrathin P-TENG. Adapted with permission from [103], Copyright © 2015, American Chemical Society.

As vast amounts of acoustic energy (e.g., from talking, traffic noise, and music) are normally not harvested, Wang group developed an ultrathin and rollable P-TENG for harvesting sound wave energy [103], which is capable of delivering a maximum power density of 121 mW·m^−2^ (volume density of approx. 968 W·m^−3^) at 800 kΩ under a sound pressure of 117 dB_SPL_ (Figure 11). The ultrathin P-TENG with a multilayered structure was composed of several thin film materials. Paper was selected as the structural backbone of the TENG (coated with copper, thereby working as an electrification layer) to generate triboelectric charges upon contact with a PTFE membrane. This device can be applied in many scenarios to harvest recycled acoustic mechanical energy from music playing and phone conversations. Compared with traditional acoustic energy harvester, which have a resonance cavity, the presented P-TENG is ultrathin, flexible and rollable. In addition, the ultrathin TENG is fabricated with low-cost, lightweight, and biodegradable paper materials and have a simple structure. The concept and design presented in [103] can be extensively applied to other energy-harvesting or sensing devices, such as wearable and flexible electronics, military surveillance equipment, equipment to reduce jet engine noise, low-cost implantable human ear, and wireless technology applications.

**Figure 12:**
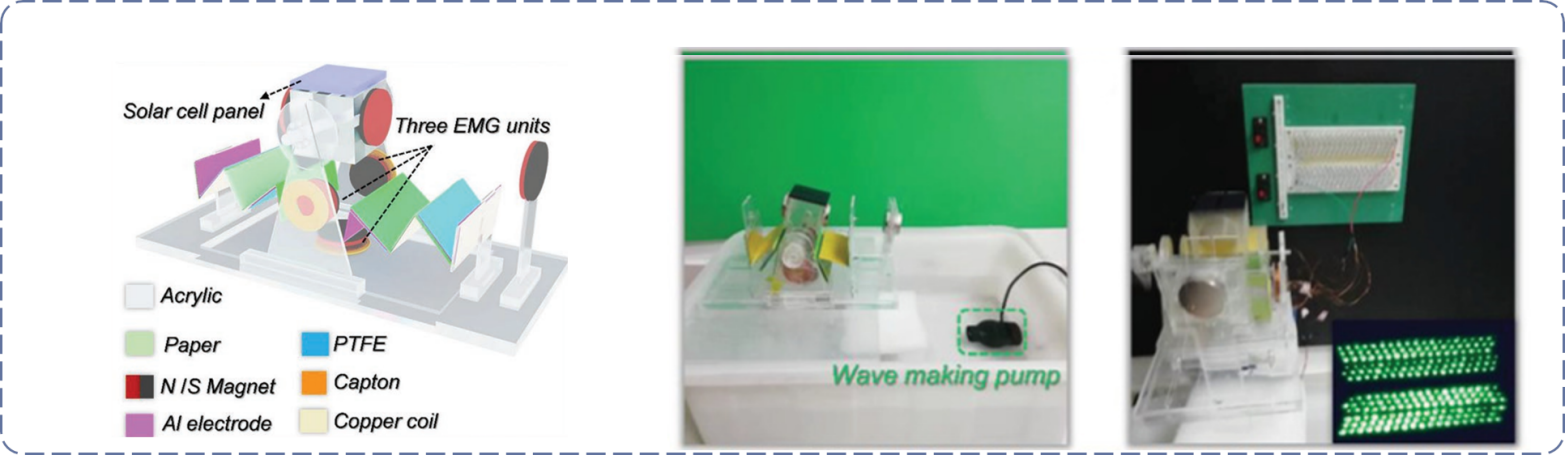
Harvesting water wave energy with a hybrid generator [163] Copyright © 2019 WILEY‐VCH Verlag GmbH & Co. KGaA, Weinheim. Adapted with permission from Hongmei Yang et al., “A Nonencapsulative Pendulum‐Like Paper–Based Hybrid Nanogenerator for Energy Harvesting”, Advanced Energy Materials, John Wiley and Sons.

Compared with the structure of 2D patterns, P-TENGs combining origami and kirigami structures are tridimensional and have more flexible structures and, therefore, more application possibilities. As TENGs are more efficient to harvest low-frequency water wave energy, robustness and packaging are of great importance for these applications. Yang et al. [163] designed a non-encapsulated pendulum-like paper-based hybrid nanogenerator to harvest water wave energy. The basic structure of the designed hybrid generator is presented in Figure 12, in which there are two zigzag multilayered P-TENG units with four basic TENGs operating in contact–separation mode on both sides of the mover. P-TENGs operating in soft–hard hybrid mode allows a highly close contact compared with the acrylic-based device. The acrylic plate can further strengthen the toughness of the paper structures and of the zigzag multilayered P-TENG, which can be considered as a spring oscillator. This unique structure revealed superior robustness and a maximum peak power value of up to 22.5 mW of the zigzag multilayered TENGs was obtained. With the two zigzag multilayered P-TENG superimposed together in parallel, *I*_sc_ increased to 41.3 µA, while *V*_oc_ decreased to 567 V and *Q*_tr_ increased to 551 nC. The charging capability of a 47 µF capacitor by TENGs and EMG was also compared by harvesting mechanical energy from water wave in [163]. Finally, the fabricated device effectively converted the water wave energy into electricity and is able to power more than 220 LEDs, revealing the potential applications in blue energy. The unique structure design of the proposed zigzag multilayered P-TENG broadens the available working frequency ranges by combining multiple modes, avoids the influence on the performance caused by harsh underwater environmental conditions, and reveals superior robustness.

Xia et al. reported a wearable P-TENG for harvesting mechanical energy from the human body [165]. In the study, X-shaped P-TENGs were assembled in cut-paper and origami-paper architectures and, thereby, were capable of providing two working patterns: cut paper and origami paper. The maximum values obtained for X-shaped P-TENGs were: *V*_oc_ (approx. 326 V), *I*_sc_ (approx. 45 μA), and power density (542.22 µW·m^−2^). The number of X-shaped units influences the P-TENG output. The *I*_sc_ value increased with an increase in the number of connected working units, whereas *V*_oc_ decreased owing to the voltage cancelation induced by different working modes. In order to enhance the voltage output, multiple full-wave rectifier bridges were employed in order to connect to each working unit for lighting up 101 high-power blue LEDs. Aside from harvesting energy in multiple directions, the X-shaped P-TENGs can also be stretched and bent due to the special geometry design. The force applied to the device is multi-directional and consistent with the motion of the human body, which allows mechanical energy harvesting from human elbow motions. This straightforward, affordable, multifunctional, and portable P-TENG device seems to enable a sustainable power supply for wearable and flexible electronic systems.

## Conclusion

The invention of paper and the discovery of triboelectrification phenomenon are both over 2000 years. Paper is by far one of the most inexpensive and widely used flexible material in daily life. The main advantages of using paper over flexible plastic substrates are the low cost, recyclability, and common use. Paper is flexible and can be easily folded or bent to form 3D structures without causing structural damage. This allows versatile structure designs and makes paper quite suitable for applications in mechanical energy-harvesting devices, such as TENGs. Triboelectrification is the basis of TENGs and it can be explained by an electron-cloud potential-well model and by an electron-emission-dominated charge-transfer mechanism. Therefore, the electrical properties of paper are of vital importance to the performance of TENG devices. Different processes and materials can be applied to paper-based devices. Conductive materials are usually evaporated, deposited, or printed on the paper. By introducing additional functional groups one can also convert the polarity of the paper to achieve a high-performance TENG. Vacuum filtration offers another fast and simple process to fabricate cellulose nanofiber-based TENG. In summary, P-TENGs can combine the merits of lightweight, low cost and flexibility and they exhibit great application potentials in self-powered sensing devices, human–machine interaction, electrochemistry, and energy harvesting.

The use of paper can also be extended to applications such as electronic displays, sensors, and smart packaging. However, low-cost flexible electronics not only require an inexpensive paper substrate, but also a fast and low-cost manufacturing process and the use of inexpensive materials. It can also be applicable to mass production if an appropriate printing technology is used. It is expected that P-TENGs can be readily integrated with other paper-based electronic devices including sensors, microfluidics, and energy storage systems. We have seen a tremendous progress, in recent years, in the field of P-TENGs, which will improve the efficiency of self-powered sensing and energy harvesting devices. However, further progress is needed in the field of electronic materials in terms of stability, performance, processability, fabrication techniques, and methods for making electronic device applications more feasible and accessible.
